# Genetic dissection of a cell-autonomous neurodegenerative disorder: lessons learned from mouse models of Niemann-Pick disease type C

**DOI:** 10.1242/dmm.012385

**Published:** 2013-08-01

**Authors:** Manuel E. Lopez, Matthew P. Scott

**Affiliations:** 1 Departments of Developmental Biology, Genetics, and Bioengineering, Howard Hughes Medical Institute, Stanford University School of Medicine, Clark Center W200, 318 Campus Drive, Stanford, CA 94305-5439, USA

## Abstract

Understanding neurodegenerative disease progression and its treatment requires the systematic characterization and manipulation of relevant cell types and molecular pathways. The neurodegenerative lysosomal storage disorder Niemann-Pick disease type C (NPC) is highly amenable to genetic approaches that allow exploration of the disease biology at the organismal, cellular and molecular level. Although NPC is a rare disease, genetic analysis of the associated neuropathology promises to provide insight into the logic of disease neural circuitry, selective neuron vulnerability and neural-glial interactions. The ability to control the disorder cell-autonomously and in naturally occurring spontaneous animal models that recapitulate many aspects of the human disease allows for an unparalleled dissection of the disease neurobiology *in vivo*. Here, we review progress in mouse-model-based studies of NPC disease, specifically focusing on the subtype that is caused by a deficiency in NPC1, a sterol-binding late endosomal membrane protein involved in lipid trafficking. We also discuss recent findings and future directions in NPC disease research that are pertinent to understanding the cellular and molecular mechanisms underlying neurodegeneration in general.

## Introduction

Worldwide, millions of new cases of neurodegenerative disease are reported every year (Meikle et al., 1999; Van Den Eeden et al., 2003; Logroscino et al., 2008; Mayeux and Stern, 2012). Affected individuals typically experience gradual disease progression and require increasing levels of health service and supportive care, which places long-term strain on the individuals and their families. Although treatments to manage the symptoms of neurodegenerative disease are available, there is an urgent need for mechanism-based treatments. Unfortunately, the underlying mechanisms of neurodegeneration and the pathways that can be manipulated to control disease are not well understood. Genetics offers an excellent tool for research and, in reality, it is the rare inheritable forms of the more common age-related dementia and motor disorders – i.e. Alzheimer’s disease (AD), Parkinson’s disease (PD) and amyotrophic lateral sclerosis (ALS) – that researchers attempt to engineer animal models of and rely on for biological insight (Wong et al., 2002). However, these age-related diseases are predominantly idiopathic and various genetic plus environmental factors contribute to overall risk. The lack of genetic *in vivo* models that can truly recapitulate the full complexity of these disorders prevents in-depth studies.

As our understanding of the biology of these diseases progresses, many parallels between different neurodegenerative disorders are becoming apparent. Neurodegenerative lysosomal storage diseases (LSDs) are rare inborn metabolic disorders that are usually caused by a gene defect that leads to deficiency of a particular lysosomal enzyme. Interestingly, gene mutations associated with LSDs, notably those in the glucocerebrosidase gene (*GBA*), have been identified as genetic risk factors for age-related disorders. *GBA* mutations cause Gaucher disease, the most common disorder among LSDs, which is linked to PD (Mazzulli et al., 2011). Mutations in *ATP13A2*, known to cause a form of juvenile Parkinsonism, also cause the LSD neuronal ceroid lipofuscinoses (NCL) (Bras et al., 2012; Dehay et al., 2012), which has a PD-related spontaneous dog model (Wöhlke et al., 2011). The increasing recognition that defects in lysosome-related pathways might underlie age-related dementia and motor neuron disease neuropathology (Nixon et al., 2008) provides incentive to thoroughly investigate the biology of LSDs.

In this Review we highlight recent advances in exploring the pathology of a rare (estimated incidence of ∼1 in 120,000–150,000 live births) neurodegenerative LSD, Niemann-Pick disease type C (NPC). NPC causes dementia in children and adults born with an autosomal recessive mutation in either of two genes, *NPC1* or *NPC2*. NPC, which is informally referred to as ‘childhood Alzheimer’s’, shares several mechanistic and biomarker similarities with AD (Reddy et al., 2006; Cologna et al., 2012). These include altered amyloid precursor protein (APP) processing and the presence of neurofibrillary tangles (Kaye, 2011). Unlike AD, NPC and other LSDs have naturally occurring mammalian models, which can be genetically manipulated and have proven to be highly useful for testing therapies (Haskins et al., 2006; Farfel-Becker et al., 2011). There is mounting evidence to suggest that NPC caused by mutations in *NPC1* can be modeled in a cell-autonomous fashion. We posit that this unique feature facilitates a detailed exploration of the underlying disease biology, which will ultimately enhance our understanding of general neurodegenerative processes.

## Clinical, genetic and biochemical features of NPC

In the early 1900s, a pediatrician named Albert Niemann reported the occurrence in an infant of a newly identified metabolic storage disorder that resembled Gaucher disease (Niemann, 1914). Physician Ludwig Pick later distinguished this unknown disease from other recognized metabolic storage disorders (Pick, 1926). Individuals with Niemann-Pick disease, as the cases were commonly referred to, showed signs of sphingomyelin storage in cells and foamy lipid deposits in body tissues, with varying degrees of organomegaly and neurological symptoms. Based on variability in clinical presentation, Niemann-Pick disease was subdivided into types A, B and C (Pentchev, 2004). In the 1960s, Niemann-Pick types A and B were identified as variants of acid sphingomyelinase deficiencies: both forms are caused by mutations in the gene encoding the lysosomal enzyme sphingomyelin phosphodiesterase 1 (SMPD1). Intriguingly, a mutation in SMPD1 has recently been described as a previously unknown risk factor for PD (Gan-Or et al., 2013). This finding further suggests that defects in lysosome-related pathways might underlie the pathology of more common disorders.

The genetic cause of Niemann-Pick type C was not discovered until the late 1990s (Loftus et al., 1997). Prior to this discovery, several spontaneous mouse models of NPC had been identified. These models played key roles in advancing our understanding of the disease. One such model was identified as a laboratory colony of BALB/c mice, whose progeny developed progressive ataxia while juvenile (3–8 weeks old), and demonstrated weight decline and early death as young adults (Morris et al., 1982). The cells of these mice exhibited a distinct cholesterol-storage disorder, and a subsequent *in vitro* survey of cell lines of human metabolic storage disorders revealed that NPC patient fibroblasts display a similar cholesterol-storage phenotype (Pentchev et al., 1986). The *NPC1* gene was subsequently identified by using an integrated human-mouse positional candidate approach (Loftus et al., 1997). It is now known that mutations that lead to partial deficiency or complete loss of function of NPC1, a 13-transmembrane endosomal cholesterol-binding protein, account for 95% of NPC cases. Defects in NPC2, a secreted cholesterol-binding protein that is believed to interact with NPC1, account for the remaining 5% of NPC cases (Naureckiene et al., 2000; Sleat et al., 2004; Cheruku et al., 2006; Deffieu and Pfeffer, 2011).

**Fig. 1.**
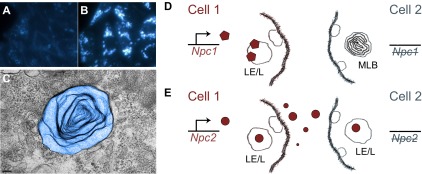
**Comparison of cell-autonomous and non-cell-autonomous rescue of the lipid-storage defect in NPC disease.** (A,B) The severe intracellular accumulation of unesterified cholesterol caused by the loss of NPC1 function is demonstrated here by comparing filipin-stained (blue) (A) wild-type K1 CHO cells and (B) *Npc1* mutant M12 CHO cells. Abundant multivesicular structures and multilamellar bodies (MLBs) accompany the storage disorder. (C) A transmission electron microscope image of a single elaborate MLB (pseudo color) is shown. (D,E) Although loss of either NPC1 or NPC2 function elicits a nearly identical cellular phenotype in NPC, (D) NPC1, a non-secreted late endosomal/lysosomal (LE/L) membrane protein, acts cell-autonomously, whereas (E) NPC2, a secreted cholesterol-binding protein, acts non-cell-autonomously. Thereby, production of NPC1 in a cell (D; cell 1) cannot correct the storage defect, depicted here by an MLB, in a neighboring NPC1-deficient cell (D; cell 2). By contrast, an NPC2-producing cell (E; cell 1) can rescue the storage defect in an NPC2-deficient cell, indicated by the absence of an MLB (E; cell 2).

The function of NPC1 remains unclear. However, biochemical studies have shown that the protein binds sterols, particularly oxysterols and cholesterol (Ohgami et al., 2004; Infante et al., 2008; Kwon et al., 2009). On this basis, NPC1 is hypothesized to be required for cholesterol egress from the lysosome. In line with this hypothesis, cells that are deficient in NPC1 accumulate a wide array of lipids, including cholesterol (Fig. 1A,B) and sphingomyelin within the endocytic system (Lloyd-Evans et al., 2008). Loss of NPC1 function also renders a cell unable to sense or effectively utilize exogenously derived cholesterol (Reddy et al., 2006; Kulinski and Vance, 2007). Thus, loss of NPC1 causes lipidosis and defects in cholesterol homeostasis.

NPC1 might act as a transporter to exchange lipid molecules between cellular compartments, to facilitate the integration of lipids into membranes, or to promote the sorting of lipids through budding and fusion of membranes. Indeed, in the absence of NPC1, endosomal organelle transport is notably perturbed (Ko et al., 2001) and multilamellar vesicle bodies abound (Fig. 1C) (Blom et al., 2003; Liao et al., 2007; Tang et al., 2009). A structural and functional comparison with related proteins, such as the membrane cholesterol transporter NPC1L1 (Smith and Levitan, 2007; Krishnan et al., 2012), or even distant relatives with conserved domains, such as the sterol-sensing domain of the Hedgehog morphogen receptor Patched (Hausmann et al., 2009), could ultimately lead to a better understanding of the molecular mechanism of action of NPC1.

What is clear about the NPC1 protein is that it is not secreted or transmitted across membranes. Thus, the loss of NPC1 function cannot be corrected by the provision of NPC1 by a neighboring cell (Fig. 1D). In contrast, the exogenous provision of NPC2 can correct the NPC lipid storage defect in cells that lack NPC2 (Fig. 1E) (Naureckiene et al., 2000). Although the cell-autonomous function of NPC1 limits the application of non-autonomous therapeutics, such as enzyme replacement therapy (Vanier, 2010), the ability to control the disorder cell-autonomously by providing NPC1 function to specific cell or tissue types enables the identification of cells that are important for neurodegenerative disease progression and, potentially, for recovery. Other useful treatments, such as substrate reduction therapies (Nietupski et al., 2012), might then be appropriately targeted to these crucial cells.

## Cell-autonomous neuronal toxicity in NPC: evidence from mouse models

A detailed understanding of what promotes cell-autonomous and non-autonomous neuron toxicity is crucial to understanding neurodegenerative disease pathogenesis. Most studies of neurodegeneration in NPC have focused on one population of highly affected cells, the Purkinje neurons of the cerebellum. However, the disease is systemic, affecting visceral endocrine tissue such as the liver as well as glial cells and neurons throughout the CNS. In disorders involving non-cell-autonomous neuronal toxicity, glial defects as well as invading toxic factors generated elsewhere in the body might cause or exacerbate CNS disease progression. These harmful cells or toxins can be targets for treatment (Ilieva et al., 2009). In disorders in which glial and immune cells contribute less to disease pathogenesis, targeting these cells might decrease the effectiveness of a treatment, because glia can have a high endocytic or phagocytic capacity for drug compounds. In different disease cases, both cell-autonomous and non-autonomous mechanisms of neurodegeneration are suspected to be present. However, autonomous and non-autonomous neurodegenerative disease mechanisms have proven difficult to tease apart and, prior to studies of NPC, a disease predominantly characterized by cell-autonomous neuronal toxicity had not been modeled. The NPC1 disease animal models, combined with cell- or tissue-specific rescue experiments, might be useful for comparing and contrasting neurodegenerative disease pathways in order to discover universal or distinct mechanisms. Thereby, NPC serves as the prototype model for cell-autonomous toxicity in a neurodegenerative disease.

Multiple complementary studies support the occurrence of cell-autonomous neurodegeneration in NPC1-deficient mice. An early NPC1 rescue study using a prion-promoter-driven *Npc1* transgene (Loftus et al., 2002) suggested that visceral tissue disease does not affect the progression of the disease in the brain. Transgene prion-promoter-directed expression, which reduced brain pathology, was widespread and not limited to neurons in the CNS. However, in mice that showed continued visceral disease progression, no neurodegeneration could be observed. This led to the conclusion that the visceral disease pathology does not impact CNS degeneration. A later study using a highly targetable Tet-inducible *Npc1* transgene further demonstrated that correction of visceral tissue pathology alone does not alter CNS disease progression (Lopez et al., 2011). Thus, CNS neurodegeneration in the NPC mouse model occurs independently of disease progression elsewhere in the body. This seems to be equally true for humans: in clinical studies, bone marrow and liver transplantation in NPC1-deficient patients altered bone marrow and liver tissue pathology but did not affect neurological signs and symptoms (Patterson, 1993; Hsu et al., 1999).

**Fig. 2.**
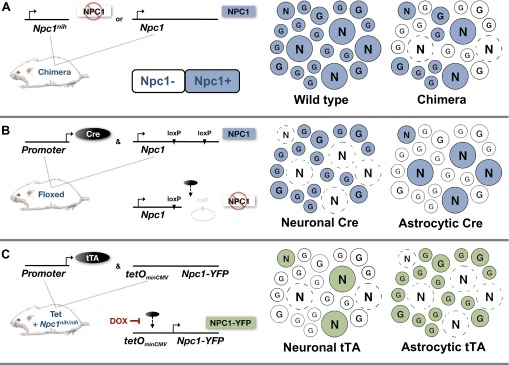
**Mouse models of cell-autonomous neuron death and survival in NPC disease.** Multiple independent studies using NPC1-deficient mouse models of the disease demonstrate that NPC1 function in neurons is necessary and sufficient to affect neurodegeneration. Mosaic depictions of the various experimental scenarios are shown. N represents neuronal cells and G represents astroglia. Dashed circles represent dead neurons and colored circles are positive for NPC1. (A) Chimeric mice with wild-type and *Npc1^nih^* mutant cells provided initial evidence for the cell-autonomous death of neurons *in vivo* (Ko et al., 2005). In these mice, *Npc1^+^* neurons survived among a mixed *Npc1^+^* and *Npc1^−^* glial population but *Npc1^−^* neurons did not survive. (B) *Npc1* conditional knockout ‘floxed’ mice (in which *Npc1* is knocked out by induction of Cre-mediated recombination) were then used to show that deletion of the *Npc1* gene in astrocytes does not contribute to neuron loss, whereas deletion of the *Npc1* gene in neurons does (Yu et al., 2011). (C) Experiments using NPC1-deficient mice (*Npc1^nih/nih^*) engineered for conditional rescue using a Tet-inducible *Npc1-YFP* transgene further demonstrated cell-autonomous survival of neurons (Lopez et al., 2011). In this study, the subset of neurons that produced NPC1-YFP in an otherwise NPC1-deficient animal survived early disease progression.

Studies focusing on the death of Purkinje neurons in NPC mice pinpointed a neuron-intrinsic cell-autonomous phenotype. In an elegant chimeric mouse experiment (Fig. 2A) that created a system in which the cerebellum contained a mixed population of normal (*Npc1^+^*) and NPC1-deficient (*Npc1^−/−^*) cells, *Npc1^−/−^* cerebellar Purkinje neurons died, whereas NPC1-producing *Npc1^+^* Purkinje neurons persisted (Ko et al., 2005). This experimental system made it possible to test whether wild-type glia, astrocytes and particularly microglia can rescue neurons, or whether neurons die regardless of the presence or absence of NPC1 function in adjacent glia. The results of this investigation showed that *Npc1^+^* glia are not able to rescue *Npc1^−/−^* neurons, and, conversely, *Npc1^+^* neurons are not damaged via proximity to *Npc1^−/−^* glia. Importantly, both *Npc1^−/−^* and *Npc1^+^* microglia accumulated selectively near dead or dying neurons. No gross difference in morphology or behavior was noted for reactive astrocytes and microglia with or without NPC1, suggesting that NPC1 normal and deficient glia act similarly when exposed to the same environment. It was concluded that neuron-intrinsic mechanisms are involved in neuron death in NPC disease. In turn, this cell-autonomous neuron injury exerts non-cell-autonomous effects that attract inflammatory glia, which then target dead or dying neurons.

The lack of neuron toxicity due to diseased astrocytes was definitively demonstrated using a conditional Cre-mediated *Npc1* knockout mouse model of NPC (Fig. 2B). Animals that lacked NPC1 in astrocytes did not exhibit any gross signs of disease progression or neurological decay, despite evidence of an astrocytic cholesterol-accumulation phenotype (Yu et al., 2011). In a complementary experiment, providing functional NPC1 to astrocytes alone in an otherwise NPC1-deficient mouse did not significantly alter disease progression, despite a slight delay in astrocyte reactivity (Fig. 2C) (Lopez et al., 2011). Taken together, these studies demonstrate that astrocytes are weakly, if at all, involved in triggering or exacerbating the initial disease pathology.

Using the conditional knockout mouse model of NPC1 disease described above, it was also shown that loss of NPC1, specifically from neurons, is sufficient to cause neurodegeneration and to recapitulate neurological signs of the disease (Fig. 2B) (Elrick et al., 2010; Yu et al., 2011). Conversely, using the Tet-inducible *Npc1* mouse model, neuron-specific NPC1 in an otherwise NPC1-deficient animal was shown to be sufficient to prevent and halt local neurodegeneration, altering neurological signs in an animal with continued systemic disease progression (Fig. 2C) (Lopez et al., 2011; Lopez et al., 2012b). These complementary results, which were obtained independently using distinct experimental methods and different mouse backgrounds, provide compelling evidence for predominant neuron-autonomous mechanisms of toxicity in NPC disease.

## Dissecting NPC disease neural circuitry in mice

As outlined above, NPC disease progression can be carefully controlled with targeted neuronal NPC1 knockout and rescue. The ability to control neuron survival in a cell-autonomous, temporal and spatial manner in the NPC mouse model offers a way to address questions of disease neural circuitry and behavior. At what stage can a neurological condition be halted or reversed? Which brain regions are involved and should be targeted therapeutically? Can surviving functional neurons in a neuronal network compensate for those that are lost? It seems possible that only partial correction of neural networks would be sufficient to elicit profound beneficial effects. Indeed, chimeric *Npc1* mice in which at least 30% of Purkinje neurons are wild type do not develop severe ataxia (Ko et al., 2005). The aspects of neural circuitry that can be corrected and maintained in these mice to halt the progression of neurological signs remain unclear.

**Fig. 3.**
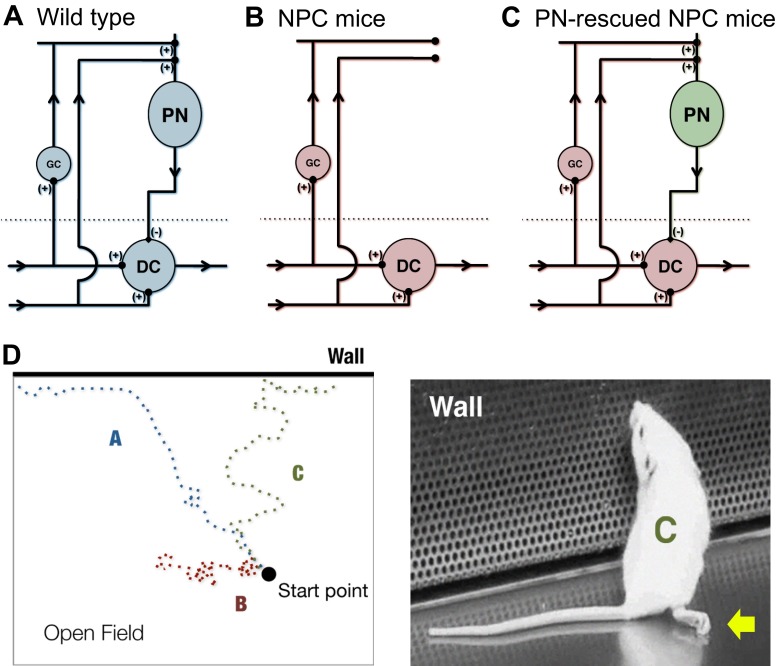
**Genetic dissection of disease neural circuits and resulting behavioral modifications.** The effects of restoring neuronal circuitry in NPC disease can be observed in a mouse model in which controlled neuron survival of discrete neuronal networks can be implemented. (A–C) Simplified circuit diagrams of the cerebellum for (A) wild-type mice, (B) NPC disease mice and (C) Purkinje neuron (PN)-rescued NPC disease mice. Compared with wild-type mice, in NPC disease mice, connectivity of PNs with granule neurons (GC) and deep cerebellar nuclei (DC) is lost, whereas PN connectivity in the cerebellar cortex is restored in PN-rescued mice (Lopez et al., 2011; Lopez et al., 2012a). (D) Even with continuous progression of severe phenotypes that affect ambulation, such as dystonia and hind limb paralysis (illustrated by arrow in right-hand mouse image), some beneficial improvements in mice activity can be observed with PN rescue (Lopez et al., 2011). Depicted here is a cartoon example of an open field test showing three tracks of mouse movement that correspond to the cerebellar conditions in panels A–C. The tracks show the difference in behavior between mice of advanced disease stages. A mouse corresponding to track B remains largely unresponsive to its environment and immobile. In comparison, a mouse corresponding to track C exhibits severe movement defects but is able to escape the open field and investigate its surroundings, similar to the wild-type mouse represented by track A.

Current attempts to decipher neural circuits rely heavily on inactivating or activating defined cell types in a normal or diseased brain (Luo et al., 2008; Tye and Deisseroth, 2012). These studies rely on the existence of a causal relationship between acute neuronal activity and behavior. Neurons, however, are much more than transmitters of electrical impulses elicited by the opening and closing of ion channels; they also serve as metabolic and endocrine centers. For example, Purkinje neurons produce neurosteroids and secrete morphogens capable of tissue remodeling and modulating glial behavior (Dahmane and Ruiz i Altaba, 1999; Sakamoto et al., 2001; Bouslama-Oueghlani et al., 2012). Thus, controlling the neuronal defect and subsequent cell-autonomous survival or death of discrete neurons in a diseased animal offers a powerful tool to investigate the developmental and behavioral effects of restoring or maintaining normal aspects of neuronal network function in a disease setting (Fig. 3).

There are several challenges to be overcome in order to be able to interpret results of neural circuit rescue. First, discrete neurons must be effectively targeted. Viral-mediated delivery and transduction, a commonly used method in optogenetic investigations of neural circuits, can limit expression to the injection site or spread rescue through neuronal connections, depending on the design and capacity of the virus (Tye and Deisseroth, 2012). Adenovirus delivery of recombinant NPC1 in the mouse brain has already been attempted (Paul et al., 2005). To target discrete neuronal populations, it might be more practical to deliver a neuronally driven transcription factor, such as tetracycline-sensitive transactivator (tTA), which can be applied in the existing Tet-inducible *Npc1* transgenic mouse, or Cre in the conditional *Npc1* knockout mouse model of NPC. Second, it is necessary to develop behavioral assays that can detect altered phenotypes. For example, Purkinje-neuron-specific rescued mice exhibit significant but temporary improvements in various disease progression parameters, such as weight and survival, but continue to have motor deficits (Lopez et al., 2011). One explanation could be the persistence of dystonic features and hind limb paralysis, which have been shown to progress independently of cerebellar involvement (Fig. 3) (Lopez et al., 2011). As a result, Purkinje-neuron-rescued mice are expected to perform poorly on rotor rod or similarly automated tests of motor skills. Other types of behavioral recordings would then be required to more accurately identify differences. The continued degeneration of other brain regions and timing of rescue would also need to be taken into account (Lopez et al., 2011; Lopez et al., 2012b).

Another concern for neuron-specific studies in NPC mouse models is the potential affect of non-cell-autonomous factors on neuronal function. For example, diseased glia or other cell types could fail to support proper neuron function and, ultimately, their survival. Despite the evidence for cell-autonomous toxicity described above, a role for non-cell-autonomous toxicity has not been ruled out. In fact, non-cell-autonomous toxicity has been widely suggested to contribute substantially to disease pathogenesis of other LSDs. Recently, using a conditional Cre-mediated deletion of the sulfatase-modifying factor 1 gene (*Sumf1*), which causes the LSD mucosulfatidosis, researchers were able to demonstrate that SUMF1-deficient astrocytes failed to support function and survival of neurons in a wild-type mouse (Di Malta et al., 2012). However, the authors did acknowledge evidence demonstrating an earlier and more severe cell-autonomous degenerative phenotype when *Sumf1* deletion was limited to neurons. This indicates that non-cell-autonomous and autonomous processes might combine in varying degrees and at various times to contribute to a neurodegenerative disorder. For NPC, the initial disease severity seems to be driven mainly by cell-autonomous factors. Whether neuron rescue alone can completely correct the disorder is uncertain because the criteria for a fully rescued or recovered state in mice is yet undetermined.

Neuronal rescue could delay early progression of the disease, but both neuronal and glial correction might ultimately be required for complete rescue. Studies using an *Npc1* transgene whose expression is driven by a neuron-specific enolase promoter allowed animal survival for more than a year, representing a reported >fivefold improvement in survival age (Borbon et al., 2012; Erickson, 2013). However, neurological defects were still observed. The possibility exists that not all critical neurons were targeted. Many ‘pan-neuronal’ drivers do not show the robust ubiquitous expression desired and often have variegated and partly silenced expression patterns (Lopez et al., 2011). Different studies might produce different results owing to the different expression patterns of the promoter and enhancer control elements used to drive cell-type-limited transcription. An alternative explanation is that non-cell-autonomous mechanisms exert greater influence with age. A recent study, using Cre-mediated *Npc1* knockout mice, demonstrated a requirement for the NPC1 protein in oligodendrocytes for appropriate formation and maintenance of myelin (Yu and Lieberman, 2013). The data suggest that, although loss of NPC1 in neurons alone leads to severe early myelin defects, loss of NPC1 in oligodendrocytes leads to the eventual loss of myelin and secondary neuron degeneration in older mice. What biological changes might elicit an age-related glial effect? Can neuron-corrected NPC1-deficient mice become a useful model for exploring non-cell-autonomous affects on neuronal circuit function? These are intriguing questions that warrant further investigation.

## Delineating a roadmap of NPC disease neuropathology

**Fig. 4.**
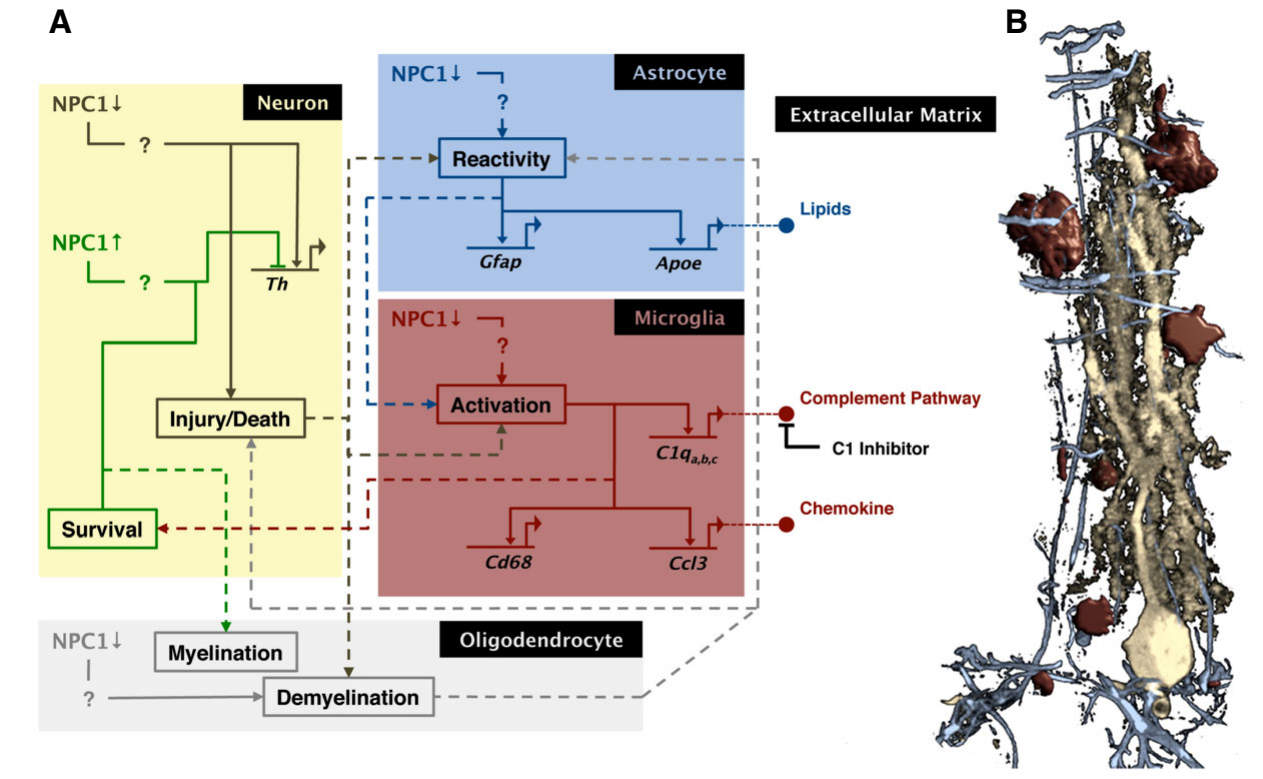
**Development of a neurodegenerative interaction map.** Cell-autonomous control of neuron rescue allows for the generation of a detailed cell-cell and molecular interaction network for the development of neurodegeneration in complex tissue. As studies add to the understanding of disease biology, a highly detailed neurodegenerative regulatory network will gradually be drawn, which ultimately could be used to determine the outcome of an intervention. (A) Illustrated here is a tentative roadmap of the pathology of cerebellar Purkinje neuron degeneration in NPC that is based on observed changes in cellular activity, gene expression pattern and protein localization in mice with conditional knockout and conditional rescue of NPC1 (Lopez et al., 2011; Yu et al., 2011; Lopez et al., 2012b; Lopez et al., 2012a; Yu and Lieberman, 2013). For simplicity, the current map only shows the following genes: *Th* (tyrosine hydroxylase), *Gfap* (glial fibrillary acidic protein), *Apoe* (apolipoprotein E), *C1q* (complement component 1, q subcomponent), *Ccl3* (chemokines C–C motif ligand 3), and *Cd68* (or macrosialin, a lysosomal membrane family protein specifically expressed by phagocytes). Solid lines represent possible intracellular pathways, and dotted lines depict intercellular interactions. The effect of NPC1 loss (brown lines) or NPC1 rescue (green lines) can be traced from the neuron. With the exception of brown and green lines, color of lines corresponds to processes in or from a particular cell type: gray, oligodendrocytes; red, microglia; blue, astrocyte. Question marks in the pathways emphasize the need to resolve the mechanisms of action further. The illustration was generated with the aid of BioTapestry software. (B) Colored regions correspond to the cells shown in a rendered confocal image of a Purkinje neuron from the cerebellar cortex of a diseased mouse. This image serves to emphasize the complexity of cell-cell interactions that occur during the disease pathology and therefore the need to assign pathways to the correct cell. Neuron is stained with Calbindin-D28K (yellow), astrocytes with GFAP (blue), and microglia with CD68 (red). Oligodendrocytes are not shown.

The ability to control neurodegeneration genetically, in a cell-specific and temporal fashion, coupled with advanced gene expression profiling and proteomic analyses, allows the possibility of delineating a regulatory network for neurodegeneration. Conceptually, the sea urchin embryonic gene regulatory network offers an excellent example of successful large-scale mapping of integrated regulatory information in the context of organismal development (Davidson et al., 2002; McClay, 2011). Focused cell and molecular manipulations of the sea urchin embryo, combined with detailed profiling of global and local gene expression changes at various stages of the embryo, led to the identification of key developmental regulatory elements. The resulting gene regulatory ‘roadmap’ has facilitated a greater understanding of the cell circuitry and regulation of early development, serving as a blueprint to decipher the similarities and differences among evolutionary divergent systems. The network is also useful as a visible template that can be questioned, countered, modified and enhanced based on new findings. Similarly, the cell circuitry and regulation of neurodegeneration in NPC disease mice could be studied broadly with genomic, proteomic and other approaches coupled with fine cell-specific genetic manipulations to provide insight into the control of neurodegeneration. Already, gene expression and proteomic profiles have been generated, mainly for cerebella (Liao et al., 2010; Cologna et al., 2012; Lopez et al., 2012b; Lopez et al., 2012a; Sleat et al., 2012), and a rudimentary schematic of cellular and molecular interactions *in vivo* can be drawn (Fig. 4A). In this schematic, we depict the possible linear progression of events stemming from a primary neuronal injury and leading to a secondary astrocytic and microglial response that accounts for the observed increase in complement and inflammatory pathway components (Lopez et al., 2012a; Lopez et al., 2012b).

A detailed roadmap of NPC disease neuropathology in mice should help to identify pathways conserved in humans that can mitigate disease and be successfully targeted by biopharmaceutical approaches. However, creating a roadmap of the disease from mouse data is not a simple task. Further profiling, determination of the timing of appearance of molecular components, and careful analysis of the localization of these components is required for the accurate construction and assignment of interactions to appropriate cell types or CNS regions (Fig. 4B). Global profiling of gene, protein or metabolic changes alone is not adequate to identify critical mechanisms. In the absence of localization and activity studies, connections between cells and interactions within each component of the network in complex tissues cannot be inferred. Facing the challenge and delineating the neurodegenerative and neuron rescue pathways in NPC genetic animal models might provide leads that are relevant to the pathology of other neurodegenerative disorders. Such explorative studies could also uncover factors that influence selective neuron vulnerability.

## Exploring selective neuron vulnerability

The ultimate pathological event in all neurodegenerative disorders is neuronal cell loss. However, not all neurons are equally vulnerable to each disease. Despite the broad and widespread presence of potential disease-causing factors, only a subset of neuron classes or distinct brain regions degenerate, and degeneration occurs at varying rates (Ilieva et al., 2009; Saxena and Caroni, 2011). This selective neuronal vulnerability is a major phenomenon of neurodegenerative disorders but is not well understood. In mice with NPC disease, the Purkinje neurons of the cerebellum or thalamic neurons seem to be particularly vulnerable to loss of NPC genes, but the reasons behind this are unknown (Sarna et al., 2003; Lopez et al., 2011). In light of the involvement of NPC proteins in intracellular trafficking and lipid accumulation, one might expect motor neurons that are sensitive to transport defects (LaMonte et al., 2002), or hippocampal neurons with potentially greater lipid accumulation defects (Lopez et al., 2011), to be more susceptible. It has been proposed that selective neuron vulnerability results in part because of heterogenous injury to supporting glial cells (Ilieva et al., 2009; Saxena and Caroni, 2011). Although data to support this argument can be found in disease models that exhibit non-autonomous toxicity, e.g. SOD1-linked ALS (Nagai et al., 2007) and spinocerebellar ataxia 7 (Custer et al., 2006), the idea does not seem to hold true for all disorders or across different experimental scenarios.

The NPC mouse model provides an ideal neurodegenerative environment for studying neuron-intrinsic factors that mediate selective neuron vulnerability. In NPC1-deficient mice, although the lysosomal defect causes the accumulation of cytoplasmic and lysosomal inclusions, vesicle transport defects (Reid et al., 2003), mitochondrial alterations (Yu et al., 2005), accumulation of oxidative byproducts (Porter et al., 2010), and even altered immune and inflammatory signaling (Sagiv et al., 2006; Liao et al., 2010) in most cells, neurodegeneration follows a particular pattern. For example, the rate and sequence of Purkinje neuron degeneration can be predicted in the NPC mouse model, and particular Purkinje neuron populations always remain resistant to cell death (Sarna et al., 2003; Ko et al., 2005). This patterned neuron loss is not easily explained by defects in glia cells or the accumulation of stressors in particular areas but does correlate with differences in gene expression across Purkinje neurons (Sarna et al., 2003). We propose that, in a cell-autonomous neurodegenerative situation, comparing gene expression changes between susceptible and non-susceptible neuronal subsets could facilitate the identification of modifiers of neuron vulnerability. It would be interesting to determine whether neurons inherently fated to die can be reprogrammed to be as resilient as their non-vulnerable counterparts. If so, this would open up avenues of treatment for mitigating disease progression, even without treating the underlying cause.

Selective neuron vulnerability has been studied in other models of neurodegenerative disease for which neuron-intrinsic mechanisms of neurodegeneration have been implicated. For example, in the TAR-DNA binding protein 43 (TDP-43) model of frontotemporal degeneration (FTD) (Neumann et al., 2006), producing the mutant form of the protein specifically in forebrain neurons causes selective neuron degeneration in mice (Igaz et al., 2011). TDP-43 is a DNA- and RNA-binding protein that regulates expression of an array of genes and might interfere with cytoplasmic-nuclear signaling (Polymenidou et al., 2011; Tollervey et al., 2011). Despite the global production of the mutant TDP-43 protein in forebrain neurons, only dentate gyrus and deep cortical layer neurons were shown to acutely degenerate. As a result of this neuron-intrinsic control of neuronal loss, similar approaches used in the study of NPC1 can be implemented using the TDP-43 model of FTD. Mouse models for both diseases allow temporal and neuron-specific control of the disease and show reproducible patterns of selective neurodegeneration. It would be intriguing to compare and contrast the NPC1 and TDP-43 models with regards to respective cortical neuron degenerative pathways. Although the two disorders differ greatly in terms of the cause of disease and vulnerability of forebrain neurons, in some cases these neurons display a similar neuronal pathophysiology, such as the occurrence of ubiquitin inclusions (Neumann et al., 2006; Bifsha et al., 2007; Xu et al., 2011). Thus, potential stress pathways that cause neurodegeneration specifically in the TDP-43 model can be identified more precisely.

## Beyond the mouse: additional models of NPC

**Fig. 5.**
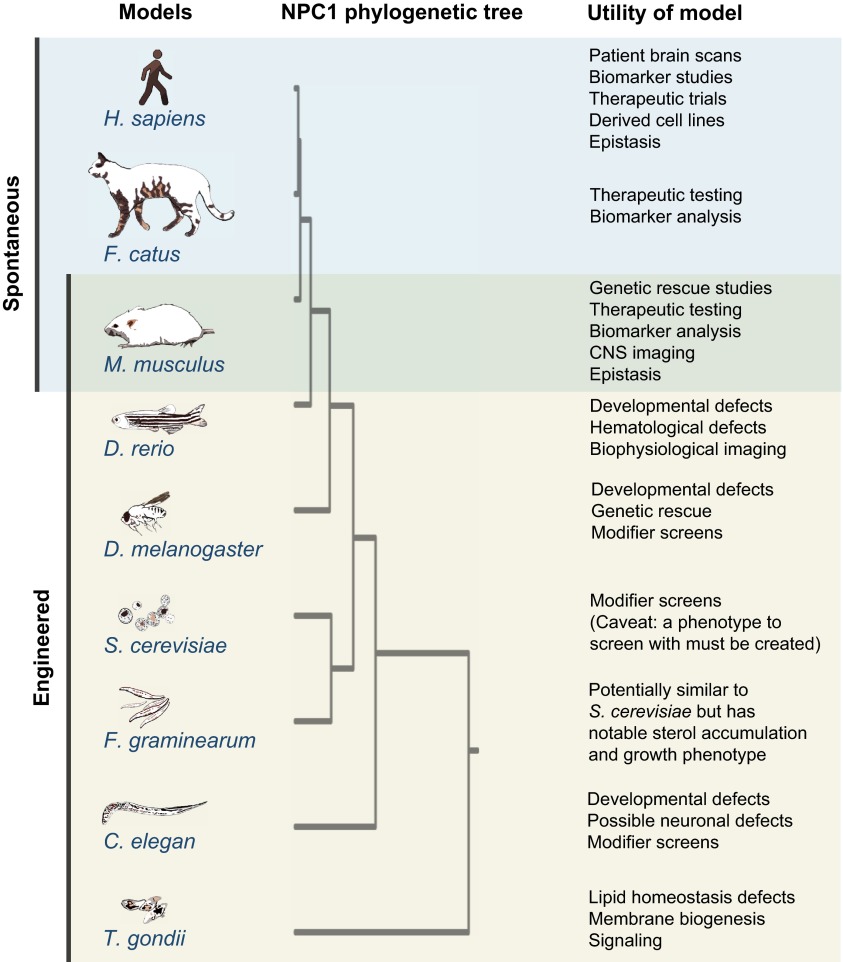
**Animal models of NPC.** The NPC1 protein is highly evolutionarily conserved among eukaryotes. Many model systems of NPC, both spontaneously derived and engineered, are available. We have listed the range of organisms in which NPC-related phenotypes have been reported (see in-text references under ‘Beyond the mouse: additional models of NPC’) and have ranked them in the order of NPC1 protein homology. Some of the current and potential uses of these model organisms for research are also listed. The phylogenetic tree was drawn using Phylogeny.fr (Dereeper et al., 2008) and the mammalian Patched protein, PTCH1, a related RND permease superfamily member and sterol-sensing-domain-containing protein (Hausmann et al., 2009), was used as an outgroup. For *C. elegans*, the NCR1 protein sequence was used. We apologize if we have failed to mention any additional organisms that have been or are currently being studied in the context of NPC.

Research on NPC is not limited to spontaneous or engineered mouse models. The NPC field also benefits from a naturally occurring cat model of the disease that is used to test proposed drug therapies (Loftus et al., 1997; Somers et al., 2003; Maue et al., 2012). Although these mammalian models are important tools for understanding the disease biology and therapeutic testing, the use of mammals is expensive and frequently inadequate for molecular, genetic and biochemical approaches. Owing to the high evolutionary conservation of the *NPC1* gene among eukaryotes, other more experimentally tractable model organisms are available (Fig. 5). A range of model organisms studied in various laboratory settings have added to our current understanding of the function of NPC1 and the mechanisms underpinning NPC.

In addition to serving as NPC models, cats happen to host the parasitic protozoan *Toxoplasma gondii* (Fig. 5), which invades the nervous system and can alter host behavior. *T. gondii* possesses TgNCR1, a sterol-sensing-domain-containing protein with sequence similarities to mammalian NPC1. Addition of the human NPC1 late endosomal localization sequence generates a chimeric TgNCR1-hNPC1 that is capable of ameliorating the NPC lipid-storage phenotypes in *Npc1* mutant Chinese hamster ovary cells (Lige et al., 2011). Parasites lacking TgNCR1 were shown to have abundant lipid storage bodies and altered membrane lipid composition. Unexpectedly, the loss of TgNCR1 induced an increased replication response, which resulted in increased virulence. The study of how TgNCR1 influences membrane lipid composition and subsequent cellular growth signaling could provide clues about the molecular and cell biology of NPC1 function.

Along with parasites, NPC1 can affect the pathogenicity of viruses. Ebola virus and its family members, which use endocytic pathways to infect a cell, require a portion of the NPC1 protein for successful integration (Carette et al., 2011). In cells lacking NPC1, the virus enters but remains trapped inside vesicles and does not replicate. Studies of Ebola infection have revealed a potential specific inhibitor of NPC1 that might be useful for exploring its molecular functions (Côté et al., 2011). Determining how Ebola and other viruses use NPC1 as a scaffold to traverse their genetic material into the cytoplasm could reveal attributes of the membrane function of NPC1.

Two commonly used laboratory model organisms, the nematode *Caenorhabditis elegans* and zebrafish *Danio rerio*, could be useful for studying developmental defects caused by lack of NPC1 (Fig. 5). *C. elegans* is an excellent model for whole-organism high-throughput chemical and molecular screening (Kwok et al., 2006). The *C. elegans* NCR-1 and NCR-2 proteins, which are thought to be involved in sterol signaling, can be functionally substituted by human NPC1 expressed in the worm, suggesting a high degree of functional conservation (Li et al., 2004; Smith and Levitan, 2007). Mammalian NPC1 also rescues the developmental defects detected in zebrafish *npc1* morphants (NPC1-deficient zebrafish generated by morpholino injection), also suggesting a high degree of functional homology between mammalian NPC1 and its counterpart in fish (Schwend et al., 2011; Louwette et al., 2013). Zebrafish offer the possibility of being able to image, in real time, cell activity *in vivo* in a vertebrate (Ahrens et al., 2012). Both model organisms require further characterization of the neurological defects caused by NPC1 protein deficiency.

Despite lacking a nervous system, yeast is a powerful single-cell model organism for genome-wide analysis of biological functions (Fig. 5). Unfortunately, loss of the yeast *NPC1* ortholog *Ncr1* does not cause a phenotype that can be easily explored by high-throughput screening assays to gain insight into NPC1 cellular pathways (Munkacsi et al., 2011). By contrast, the filamentous fungus *Fusarium graminearum* does have a discernible NPC storage phenotype upon deletion of the *NPC1* ortholog (Breakspear et al., 2011). As in yeast, the NPC1 ortholog in *F. graminearum* localizes to the vacuolar membrane, which is homologous to the lysosome. In contrast with yeast, mutant *F. graminearum* strains accumulate ergosterol (a fungal sterol), show sensitivity to ergosterol synthesis inhibitors and have a temperature-dependent reduction of growth. Tools that would enable full exploitation of *F. graminearum* as a model system are currently lacking, but this could change with technological advances in this area.

One organism that has a nervous system and reigns supreme in the world of genetic screens is *Drosophila melanogaster.* The fly model benefits from fast reproduction times and the availability of a vast number of genetic resources. Fly models of NPC1 and NPC2 have been generated (Fig. 5), revealing sterol-usage and steroid-synthesis defects (Huang et al., 2005; Fluegel et al., 2006; Phillips et al., 2008). dNPC1a, the fly homolog of the NPC1 protein, has been shown by MARCM (mosaic analysis with a repressible cell marker) clonal analysis to be required in a cell-autonomous fashion to prevent intracellular cholesterol-storage defects (Phillips et al., 2008). Moreover, neuron-specific rescue studies in flies have shown a strong neuronal requirement for dNPC1a as well as selective neuron vulnerability (Phillips et al., 2008).

In addition to model organisms, human cell lines from the differentiation of stem cells and other sources provide unique opportunities to perform cellular studies and test therapeutic compounds for neurodegenerative disease (Ordonez et al., 2012). Although these studies often do not mimic natural organs or tissues, they can be coupled with information gleamed from *in vivo* models. Of course, the greatest system for *in vivo* study is provided by affected individuals (Fig. 5). Thorough analyses of signs and symptoms along with well-designed clinical trials might help uncover biomarkers of disease progression and identify new drug targets.

## Conclusions and future perspectives

The study of rare genetic disorders, although often neglected by biopharmaceutical companies, can facilitate important discoveries in our understanding and treatment of more common idiopathic diseases. For example, investigation of homozygous familial hypercholesterolemia, a rare disease with a prevalence of 1 in 1 million, led to the discovery of the low-density lipoprotein (LDL) receptor (Goldstein and Brown, 2009). This initial finding facilitated dramatic advances in our understanding of cholesterol homeostasis, receptor-lysosome biology and the mechanism by which statins, drugs used to prevent heart attacks, lower plasma LDL cholesterol. Although NPC is a rare neurodegenerative disease, the naturally occurring and engineered genetic models of the disease show great potential for the elucidation of neurodegenerative disease biology that is translatable to humans. The lessons learned from exploring the disease in mice and other organisms could be invaluable for understanding mechanisms of neurodegeneration that are conserved or differ between organisms and among disparate neurological disorders.

Since the discovery of the *NPC1* disease gene, remarkable efforts have been made to generate genetic tools to study the neurodegenerative aspects of the disease. In mice alone, multiple targeted transgenics, chimeras, a conditional knockout, and a regulable cell-type-specific disease-rescue model have been engineered. Despite the advances made, caution should be exercised in analyzing the data generated in mouse-based studies. Altered patterns of transgene expression within a mouse population and with age can affect results (Lopez et al., 2011). In the development of future models, or the use of current NPC transgenic mice, multiple approaches can and should be used to assess correct cell and tissue targeting of a particular genetic modification. Strain differences can also account for divergent phenotypes (Liu et al., 2008). For example, *Npc1*-null mutations put into the C57BL/6 mouse strain cause a more severe early defect (leading to premature death prior to obvious neurodegeneration) than in FVB/N, Balb/c or mixed genetic backgrounds. In the C57BL/6 mouse strain, visceral tissue defects and inflammatory factors might play a more central role (Parra et al., 2011). A novel C57BL/6 mouse strain harboring a humanized mutation of NPC1 has recently been characterized and reported as more closely mimicking the signs and symptoms of the human disorder, probably because the allele is hypomorphic and disease progression is therefore slower in this strain (Maue et al., 2012). In spite of the caveats associated with mice studies, researchers should not be dissuaded from tackling the disease complexity *in vivo* using these or other model organisms. We anticipate that precise targeted manipulations and the ability to accurately trace the affected cell types will lead to a better understanding of disease pathology.

As is evident for most instances of inherited neurodegenerative disease, in NPC the causative gene factor, *NPC1*, is ubiquitously expressed, yet subsets of neurons and a few other cell types are exceptionally vulnerable to loss of NPC1 function. This root problem must be addressed by determining the intracellular events that lead to selective neuron malfunction, injury and cell death. NPC, caused by loss of NPC1 function, continues to serve as a prototype for studying cell-autonomous neurodegeneration. As a result of the cell-autonomous function of NPC1 and ability to manipulate specific populations of cells genetically, a detailed roadmap of the progression and rescue of neurodegeneration is gradually being generated. Although the remaining obstacles to fully understanding neurodegenerative disease pathology are challenging, the increasingly powerful genetic, molecular and imaging tools available support the optimistic view that a deep understanding of disease neurobiology and methods to control the disease can be obtained.

## References

[b1-0061089] Ahrens M. B. Li J. M. Orger M. B. Robson D. N. Schier A. F. Engert F. Portugues R. (2012). Brain-wide neuronal dynamics during motor adaptation in zebrafish. Nature 485, 471–477 22622571 10.1038/nature11057 PMC3618960

[b2-0061089] Bifsha P. Landry K. Ashmarina L. Durand S. Seyrantepe V. Trudel S. Quiniou C. Chemtob S. Xu Y. Gravel R. A. (2007). Altered gene expression in cells from patients with lysosomal storage disorders suggests impairment of the ubiquitin pathway. Cell Death Differ. 14, 511–523 16888648 10.1038/sj.cdd.4402013

[b3-0061089] Blom T. S. Linder M. D. Snow K. Pihko H. Hess M. W. Jokitalo E. Veckman V. Syvänen A. C. Ikonen E. (2003). Defective endocytic trafficking of NPC1 and NPC2 underlying infantile Niemann-Pick type C disease. Hum. Mol. Genet. 12, 257–272 12554680 10.1093/hmg/ddg025

[b4-0061089] Borbon I. Totenhagen J. Fiorenza M. T. Canterini S. Ke W. Trouard T. Erickson R. P. (2012). Niemann-Pick C1 mice, a model of ‘juvenile Alzheimer’s disease’, with normal gene expression in neurons and fibrillary astrocytes show long term survival and delayed neurodegeneration. J. Alzheimers Dis. 30, 875–887 22495346 10.3233/JAD-2012-120199

[b5-0061089] Bouslama-Oueghlani L. Wehrlé R. Doulazmi M. Chen X. R. Jaudon F. Lemaigre-Dubreuil Y. Rivals I. Sotelo C. Dusart I. (2012). Purkinje cell maturation participates in the control of oligodendrocyte differentiation: role of sonic hedgehog and vitronectin. PLoS ONE 7, e49015 23155445 10.1371/journal.pone.0049015 PMC3498367

[b6-0061089] Bras J. Verloes A. Schneider S. A. Mole S. E. Guerreiro R. J. (2012). Mutation of the parkinsonism gene ATP13A2 causes neuronal ceroid-lipofuscinosis. Hum. Mol. Genet. 21, 2646–2650 22388936 10.1093/hmg/dds089 PMC3363329

[b7-0061089] Breakspear A. Pasquali M. Broz K. Dong Y. Kistler H. C. (2011). Npc1 is involved in sterol trafficking in the filamentous fungus Fusarium graminearum. Fungal Genet. Biol. 48, 725–730 21397712 10.1016/j.fgb.2011.03.001

[b8-0061089] Carette J. E. Raaben M. Wong A. C. Herbert A. S. Obernosterer G. Mulherkar N. Kuehne A. I. Kranzusch P. J. Griffin A. M. Ruthel G. (2011). Ebola virus entry requires the cholesterol transporter Niemann-Pick C1. Nature 477, 340–343 21866103 10.1038/nature10348 PMC3175325

[b9-0061089] Cheruku S. R. Xu Z. Dutia R. Lobel P. Storch J. (2006). Mechanism of cholesterol transfer from the Niemann-Pick type C2 protein to model membranes supports a role in lysosomal cholesterol transport. J. Biol. Chem. 281, 31594–31604 16606609 10.1074/jbc.M602765200

[b10-0061089] Cologna S. M. Jiang X. S. Backlund P. S. Cluzeau C. V. Dail M. K. Yanjanin N. M. Siebel S. Toth C. L. Jun H. S. Wassif C. A. (2012). Quantitative proteomic analysis of Niemann-Pick disease, type C1 cerebellum identifies protein biomarkers and provides pathological insight. PLoS ONE 7, e47845 23144710 10.1371/journal.pone.0047845 PMC3483225

[b11-0061089] Côté M. Misasi J. Ren T. Bruchez A. Lee K. Filone C. M. Hensley L. Li Q. Ory D. Chandran K. (2011). Small molecule inhibitors reveal Niemann-Pick C1 is essential for Ebola virus infection. Nature 477, 344–348 21866101 10.1038/nature10380 PMC3230319

[b12-0061089] Custer S. K. Garden G. A. Gill N. Rueb U. Libby R. T. Schultz C. Guyenet S. J. Deller T. Westrum L. E. Sopher B. L. (2006). Bergmann glia expression of polyglutamine-expanded ataxin-7 produces neurodegeneration by impairing glutamate transport. Nat. Neurosci. 9, 1302–1311 16936724 10.1038/nn1750

[b13-0061089] Dahmane N. Ruiz i Altaba A. (1999). Sonic hedgehog regulates the growth and patterning of the cerebellum. Development 126, 3089–3100 10375501 10.1242/dev.126.14.3089

[b14-0061089] Davidson E. H. Rast J. P. Oliveri P. Ransick A. Calestani C. Yuh C. H. Minokawa T. Amore G. Hinman V. Arenas-Mena C. (2002). A genomic regulatory network for development. Science 295, 1669–1678 11872831 10.1126/science.1069883

[b15-0061089] Deffieu M. S. Pfeffer S. R. (2011). Niemann-Pick type C 1 function requires lumenal domain residues that mediate cholesterol-dependent NPC2 binding. Proc. Natl. Acad. Sci. USA 108, 18932–18936 22065762 10.1073/pnas.1110439108 PMC3223457

[b16-0061089] Dehay B. Ramirez A. Martinez-Vicente M. Perier C. Canron M. H. Doudnikoff E. Vital A. Vila M. Klein C. Bezard E. (2012). Loss of P-type ATPase ATP13A2/PARK9 function induces general lysosomal deficiency and leads to Parkinson disease neurodegeneration. Proc. Natl. Acad. Sci. USA 109, 9611–9616 22647602 10.1073/pnas.1112368109 PMC3386132

[b17-0061089] Dereeper A. Guignon V. Blanc G. Audic S. Buffet S. Chevenet F. Dufayard J. F. Guindon S. Lefort V. Lescot M. (2008). Phylogeny.fr: robust phylogenetic analysis for the non-specialist. Nucleic Acids Res. 36 **Web Server issue**, W465–469 18424797 10.1093/nar/gkn180 PMC2447785

[b18-0061089] Di Malta C. Fryer J. D. Settembre C. Ballabio A. (2012). Astrocyte dysfunction triggers neurodegeneration in a lysosomal storage disorder. Proc. Natl. Acad. Sci. USA 109, E2334–E2342 22826245 10.1073/pnas.1209577109 PMC3435187

[b19-0061089] Elrick M. J. Pacheco C. D. Yu T. Dadgar N. Shakkottai V. G. Ware C. Paulson H. L. Lieberman A. P. (2010). Conditional Niemann-Pick C mice demonstrate cell autonomous Purkinje cell neurodegeneration. Hum. Mol. Genet. 19, 837–847 20007718 10.1093/hmg/ddp552 PMC2816612

[b20-0061089] Erickson R. P. (2013). Current controversies in Niemann-Pick C1 disease: steroids or gangliosides; neurons or neurons and glia. J. Appl. Genet. 54, 215–224 23292954 10.1007/s13353-012-0130-0

[b21-0061089] Farfel-Becker T. Vitner E. B. Futerman A. H. (2011). Animal models for Gaucher disease research. Dis. Model. Mech. 4, 746–752 21954067 10.1242/dmm.008185 PMC3209644

[b22-0061089] Fluegel M. L. Parker T. J. Pallanck L. J. (2006). Mutations of a Drosophila NPC1 gene confer sterol and ecdysone metabolic defects. Genetics 172, 185–196 16079224 10.1534/genetics.105.046565 PMC1456146

[b23-0061089] Gan-Or Z. Ozelius L. J. Bar-Shira A. Saunders-Pullman R. Mirelman A. Kornreich R. Gana-Weisz M. Raymond D. Rozenkrantz L. Deik A. (2013). The p.L302P mutation in the lysosomal enzyme gene SMPD1 is a risk factor for Parkinson disease. Neurology 80, 1606–1610 23535491 10.1212/WNL.0b013e31828f180e PMC3662322

[b24-0061089] Goldstein J. L. Brown M. S. (2009). The LDL receptor. Arterioscler. Thromb. Vasc. Biol. 29, 431–438 19299327 10.1161/ATVBAHA.108.179564 PMC2740366

[b25-0061089] Haskins M. E. Giger U. Patterson D. F. (2006). Animal models of lysosomal storage diseases: their development and clinical relevance. In Fabry Disease: Perspectives from 5 Years of FOS (ed. Mehta A. Beck M. Sunder-Plassmann G.). Oxford: Oxford PharmaGenesis 21290677

[b26-0061089] Hausmann G. von Mering C. Basler K. (2009). The hedgehog signaling pathway: where did it come from? PLoS Biol. 7, e1000146 19564910 10.1371/journal.pbio.1000146 PMC2698682

[b27-0061089] Hsu Y. S. Hwu W. L. Huang S. F. Lu M. Y. Chen R. L. Lin D. T. Peng S. S. Lin K. H. (1999). Niemann-Pick disease type C (a cellular cholesterol lipidosis) treated by bone marrow transplantation. Bone Marrow Transplant. 24, 103–107 10435744 10.1038/sj.bmt.1701826

[b28-0061089] Huang X. Suyama K. Buchanan J. Zhu A. J. Scott M. P. (2005). A Drosophila model of the Niemann-Pick type C lysosome storage disease: dnpc1a is required for molting and sterol homeostasis. Development 132, 5115–5124 16221727 10.1242/dev.02079

[b29-0061089] Igaz L. M. Kwong L. K. Lee E. B. Chen-Plotkin A. Swanson E. Unger T. Malunda J. Xu Y. Winton M. J. Trojanowski J. Q. (2011). Dysregulation of the ALS-associated gene TDP-43 leads to neuronal death and degeneration in mice. J. Clin. Invest. 121, 726–738 21206091 10.1172/JCI44867 PMC3026736

[b30-0061089] Ilieva H. Polymenidou M. Cleveland D. W. (2009). Non-cell autonomous toxicity in neurodegenerative disorders: ALS and beyond. J. Cell Biol. 187, 761–772 19951898 10.1083/jcb.200908164 PMC2806318

[b31-0061089] Infante R. E. Abi-Mosleh L. Radhakrishnan A. Dale J. D. Brown M. S. Goldstein J. L. (2008). Purified NPC1 protein. I. Binding of cholesterol and oxysterols to a 1278-amino acid membrane protein. J. Biol. Chem. 283, 1052–1063 17989073 10.1074/jbc.M707943200

[b32-0061089] Kaye E. M. (2011). Niemann-Pick C disease: not your average lysosomal storage disease. Neurology 76, 316–317 21205672 10.1212/WNL.0b013e3182088310

[b33-0061089] Ko D. C. Gordon M. D. Jin J. Y. Scott M. P. (2001). Dynamic movements of organelles containing Niemann-Pick C1 protein: NPC1 involvement in late endocytic events. Mol. Biol. Cell 12, 601–614 11251074 10.1091/mbc.12.3.601 PMC30967

[b34-0061089] Ko D. C. Milenkovic L. Beier S. M. Manuel H. Buchanan J. Scott M. P. (2005). Cell-autonomous death of cerebellar purkinje neurons with autophagy in Niemann-Pick type C disease. PLoS Genet. 1, 81–95 16103921 10.1371/journal.pgen.0010007 PMC1183526

[b35-0061089] Krishnan A. Miller E. H. Herbert A. S. Ng M. Ndungo E. Whelan S. P. Dye J. M. Chandran K. (2012). Niemann-Pick C1 (NPC1)/NPC1-like1 chimeras define sequences critical for NPC1’s function as a flovirus entry receptor. Viruses 4, 2471–2484 23202491 10.3390/v4112471 PMC3509659

[b36-0061089] Kulinski A. Vance J. E. (2007). Lipid homeostasis and lipoprotein secretion in Niemann-Pick C1-deficient hepatocytes. J. Biol. Chem. 282, 1627–1637 17107950 10.1074/jbc.M610001200

[b37-0061089] Kwok T. C. Ricker N. Fraser R. Chan A. W. Burns A. Stanley E. F. McCourt P. Cutler S. R. Roy P. J. (2006). A small-molecule screen in C. elegans yields a new calcium channel antagonist. Nature 441, 91–95 16672971 10.1038/nature04657

[b38-0061089] Kwon H. J. Abi-Mosleh L. Wang M. L. Deisenhofer J. Goldstein J. L. Brown M. S. Infante R. E. (2009). Structure of N-terminal domain of NPC1 reveals distinct subdomains for binding and transfer of cholesterol. Cell 137, 1213–1224 19563754 10.1016/j.cell.2009.03.049 PMC2739658

[b39-0061089] LaMonte B. H. Wallace K. E. Holloway B. A. Shelly S. S. Ascaño J. Tokito M. Van Winkle T. Howland D. S. Holzbaur E. L. (2002). Disruption of dynein/dynactin inhibits axonal transport in motor neurons causing late-onset progressive degeneration. Neuron 34, 715–727 12062019 10.1016/s0896-6273(02)00696-7

[b40-0061089] Li J. Brown G. Ailion M. Lee S. Thomas J. H. (2004). NCR-1 and NCR-2, the C. elegans homologs of the human Niemann-Pick type C1 disease protein, function upstream of DAF-9 in the dauer formation pathways. Development 131, 5741–5752 15509773 10.1242/dev.01408

[b41-0061089] Liao G. Yao Y. Liu J. Yu Z. Cheung S. Xie A. Liang X. Bi X. (2007). Cholesterol accumulation is associated with lysosomal dysfunction and autophagic stress in Npc1 −/− mouse brain. Am. J. Pathol. 171, 962–975 17631520 10.2353/ajpath.2007.070052 PMC1959498

[b42-0061089] Liao G. Wen Z. Irizarry K. Huang Y. Mitsouras K. Darmani M. Leon T. Shi L. Bi X. (2010). Abnormal gene expression in cerebellum of Npc1−/− mice during postnatal development. Brain Res. 1325, 128–140 20153740 10.1016/j.brainres.2010.02.019 PMC2848886

[b43-0061089] Lige B. Romano J. D. Bandaru V. V. Ehrenman K. Levitskaya J. Sampels V. Haughey N. J. Coppens I. (2011). Deficiency of a Niemann-Pick, type C1-related protein in toxoplasma is associated with multiple lipidoses and increased pathogenicity. PLoS Pathog. 7, e1002410 22174676 10.1371/journal.ppat.1002410 PMC3234224

[b44-0061089] Liu B. Li H. Repa J. J. Turley S. D. Dietschy J. M. (2008). Genetic variations and treatments that affect the lifespan of the NPC1 mouse. J. Lipid Res. 49, 663–669 18077828 10.1194/jlr.M700525-JLR200

[b45-0061089] Lloyd-Evans E. Morgan A. J. He X. Smith D. A. Elliot-Smith E. Sillence D. J. Churchill G. C. Schuchman E. H. Galione A. Platt F. M. (2008). Niemann-Pick disease type C1 is a sphingosine storage disease that causes deregulation of lysosomal calcium. Nat. Med. 14, 1247–1255 18953351 10.1038/nm.1876

[b46-0061089] Loftus S. K. Morris J. A. Carstea E. D. Gu J. Z. Cummings C. Brown A. Ellison J. Ohno K. Rosenfeld M. A. Tagle D. A. (1997). Murine model of Niemann-Pick C disease: mutation in a cholesterol homeostasis gene. Science 277, 232–235 9211850 10.1126/science.277.5323.232

[b47-0061089] Loftus S. K. Erickson R. P. Walkley S. U. Bryant M. A. Incao A. Heidenreich R. A. Pavan W. J. (2002). Rescue of neurodegeneration in Niemann-Pick C mice by a prion-promoter-driven Npc1 cDNA transgene. Hum. Mol. Genet. 11, 3107–3114 12417532 10.1093/hmg/11.24.3107

[b48-0061089] Logroscino G. Traynor B. J. Hardiman O. Chio’ A. Couratier P. Mitchell J. D. Swingler R. J. Beghi E. EURALS (2008). Descriptive epidemiology of amyotrophic lateral sclerosis: new evidence and unsolved issues. J. Neurol. Neurosurg. Psychiatry 79, 6–11 18079297 10.1136/jnnp.2006.104828

[b49-0061089] Lopez M. E. Klein A. D. Dimbil U. J. Scott M. P. (2011). Anatomically defined neuron-based rescue of neurodegenerative Niemann-Pick type C disorder. J. Neurosci. 31, 4367–4378 21430138 10.1523/JNEUROSCI.5981-10.2011 PMC3071647

[b50-0061089] Lopez M. E. Klein A. D. Scott M. P. (2012a). Complement is dispensable for neurodegeneration in Niemann-Pick disease type C. J. Neuroinflammation 9, 216 22985423 10.1186/1742-2094-9-216 PMC3511250

[b51-0061089] Lopez M. E. Klein A. D. Hong J. Dimbil U. J. Scott M. P. (2012b). Neuronal and epithelial cell rescue resolves chronic systemic inflammation in the lipid storage disorder Niemann-Pick C. Hum. Mol. Genet. 21, 2946–2960 22493001 10.1093/hmg/dds126 PMC3373242

[b52-0061089] Louwette S. Regal L. Wittevrongel C. Thys C. Vandeweeghde G. Decuyper E. Leemans P. De Vos R. Van Geet C. Jaeken J. (2013). NPC1 defect results in abnormal platelet formation and function: studies in Niemann-Pick disease type C1 patients and zebrafish. Hum. Mol. Genet. 22, 61–73 23010472 10.1093/hmg/dds401

[b53-0061089] Luo L. Callaway E. M. Svoboda K. (2008). Genetic dissection of neural circuits. Neuron 57, 634–660 18341986 10.1016/j.neuron.2008.01.002 PMC2628815

[b54-0061089] Maue R. A. Burgess R. W. Wang B. Wooley C. M. Seburn K. L. Vanier M. T. Rogers M. A. Chang C. C. Chang T. Y. Harris B. T. (2012). A novel mouse model of Niemann-Pick type C disease carrying a D1005G-Npc1 mutation comparable to commonly observed human mutations. Hum. Mol. Genet. 21, 730–750 22048958 10.1093/hmg/ddr505 PMC3263988

[b55-0061089] Mayeux R. Stern Y. (2012). Epidemiology of Alzheimer disease. Cold Spring Harb. Perspect Med. 2012, 10.1101/cshperspect.a006239 PMC3405821 22908189

[b56-0061089] Mazzulli J. R. Xu Y. H. Sun Y. Knight A. L. McLean P. J. Caldwell G. A. Sidransky E. Grabowski G. A. Krainc D. (2011). Gaucher disease glucocerebrosidase and α-synuclein form a bidirectional pathogenic loop in synucleinopathies. Cell 146, 37–52 21700325 10.1016/j.cell.2011.06.001 PMC3132082

[b57-0061089] McClay D. R. (2011). Evolutionary crossroads in developmental biology: sea urchins. Development 138, 2639–2648 21652646 10.1242/dev.048967 PMC3109595

[b58-0061089] Meikle P. J. Hopwood J. J. Clague A. E. Carey W. F. (1999). Prevalence of lysosomal storage disorders. JAMA 281, 249–254 9918480 10.1001/jama.281.3.249

[b59-0061089] Morris M. D. Bhuvaneswaran C. Shio H. Fowler S. (1982). Lysosome lipid storage disorder in NCTR-BALB/c mice. I. Description of the disease and genetics. Am. J. Pathol. 108, 140–149 6765731 PMC1916074

[b60-0061089] Munkacsi A. B. Chen F. W. Brinkman M. A. Higaki K. Gutiérrez G. D. Chaudhari J. Layer J. V. Tong A. Bard M. Boone C. (2011). An ‘exacerbate-reverse’ strategy in yeast identifies histone deacetylase inhibition as a correction for cholesterol and sphingolipid transport defects in human Niemann-Pick type C disease. J. Biol. Chem. 286, 23842–23851 21489983 10.1074/jbc.M111.227645 PMC3129166

[b61-0061089] Nagai M. Re D. B. Nagata T. Chalazonitis A. Jessell T. M. Wichterle H. Przedborski S. (2007). Astrocytes expressing ALS-linked mutated SOD1 release factors selectively toxic to motor neurons. Nat. Neurosci. 10, 615–622 17435755 10.1038/nn1876 PMC3799799

[b62-0061089] Naureckiene S. Sleat D. E. Lackland H. Fensom A. Vanier M. T. Wattiaux R. Jadot M. Lobel P. (2000). Identification of HE1 as the second gene of Niemann-Pick C disease. Science 290, 2298–2301 11125141 10.1126/science.290.5500.2298

[b63-0061089] Neumann M. Sampathu D. M. Kwong L. K. Truax A. C. Micsenyi M. C. Chou T. T. Bruce J. Schuck T. Grossman M. Clark C. M. (2006). Ubiquitinated TDP-43 in frontotemporal lobar degeneration and amyotrophic lateral sclerosis. Science 314, 130–133 17023659 10.1126/science.1134108

[b64-0061089] Niemann A. (1914). Ein unbekanntes Krankheitsbild. Jahrbuch für Kinderheilkunde 79, 1–10

[b65-0061089] Nietupski J. B. Pacheco J. J. Chuang W. L. Maratea K. Li L. Foley J. Ashe K. M. Cooper C. G. Aerts J. M. Copeland D. P. (2012). Iminosugar-based inhibitors of glucosylceramide synthase prolong survival but paradoxically increase brain glucosylceramide levels in Niemann-Pick C mice. Mol. Genet. Metab. 105, 621–628 22366055 10.1016/j.ymgme.2012.01.020

[b66-0061089] Nixon R. A. Yang D. S. Lee J. H. (2008). Neurodegenerative lysosomal disorders: a continuum from development to late age. Autophagy 4, 590–599 18497567 10.4161/auto.6259

[b67-0061089] Ohgami N. Ko D. C. Thomas M. Scott M. P. Chang C. C. Chang T. Y. (2004). Binding between the Niemann-Pick C1 protein and a photoactivatable cholesterol analog requires a functional sterol-sensing domain. Proc. Natl. Acad. Sci. USA 101, 12473–12478 15314240 10.1073/pnas.0405255101 PMC514655

[b68-0061089] Ordonez M. P. Roberts E. A. Kidwell C. U. Yuan S. H. Plaisted W. C. Goldstein L. S. (2012). Disruption and therapeutic rescue of autophagy in a human neuronal model of Niemann Pick type C1. Hum. Mol. Genet. 21, 2651–2662 22437840 10.1093/hmg/dds090 PMC3363339

[b69-0061089] Parra J. Klein A. D. Castro J. Morales M. G. Mosqueira M. Valencia I. Cortés V. Rigotti A. Zanlungo S. (2011). Npc1 deficiency in the C57BL/6J genetic background enhances Niemann-Pick disease type C spleen pathology. Biochem. Biophys. Res. Commun. 413, 400–406 21910975 10.1016/j.bbrc.2011.08.096

[b70-0061089] Patterson M. (1993). Niemann-Pick disease type C. In GeneReviews (ed. Pagon R. A. Bird T. D. Dolan C. R. Stephens K. Adam M. P.). Seattle, WA: University of Washington, Seattle

[b71-0061089] Paul C. A. Reid P. C. Boegle A. K. Karten B. Zhang M. Jiang Z. G. Franz D. Lin L. Chang T. Y. Vance J. E. (2005). Adenovirus expressing an NPC1-GFP fusion gene corrects neuronal and nonneuronal defects associated with Niemann pick type C disease. J. Neurosci. Res. 81, 706–719 16015597 10.1002/jnr.20592

[b72-0061089] Pentchev P. G. (2004). Niemann-Pick C research from mouse to gene. Biochim. Biophys. Acta 1685, 3–7 15465420 10.1016/j.bbalip.2004.08.005

[b73-0061089] Pentchev P. G. Comly M. E. Kruth H. S. Patel S. Proestel M. Weintroub H. (1986). The cholesterol storage disorder of the mutant BALB/c mouse. A primary genetic lesion closely linked to defective esterification of exogenously derived cholesterol and its relationship to human type C Niemann-Pick disease. J. Biol. Chem. 261, 2772–2777 3949747

[b74-0061089] Phillips S. E. Woodruff E. A. 3rd Liang P. Patten M. Broadie K. (2008). Neuronal loss of Drosophila NPC1a causes cholesterol aggregation and age-progressive neurodegeneration. J. Neurosci. 28, 6569–6582 18579730 10.1523/JNEUROSCI.5529-07.2008 PMC3306184

[b75-0061089] Pick L. (1926). Der morbus gaucher und die ihm ähnlichen krankheiten (die lipoidzellige splenohepatomegalie typus Niemann und die diabetische lipoidzellenhypoplasie der milz). Ergebnisse der Inneren Medizin und Kinderheilkunde (Berlin) 29, 519–627

[b76-0061089] Polymenidou M. Lagier-Tourenne C. Hutt K. R. Huelga S. C. Moran J. Liang T. Y. Ling S. C. Sun E. Wancewicz E. Mazur C. (2011). Long pre-mRNA depletion and RNA missplicing contribute to neuronal vulnerability from loss of TDP-43. Nat. Neurosci. 14, 459–468 21358643 10.1038/nn.2779 PMC3094729

[b77-0061089] Porter F. D. Scherrer D. E. Lanier M. H. Langmade S. J. Molugu V. Gale S. E. Olzeski D. Sidhu R. Dietzen D. J. Fu R. (2010). Cholesterol oxidation products are sensitive and specific blood-based biomarkers for Niemann-Pick C1 disease. Sci. Transl. Med. 2, 56ra81 10.1126/scitranslmed.3001417 PMC3170139 21048217

[b78-0061089] Reddy J. V. Ganley I. G. Pfeffer S. R. (2006). Clues to neuro-degeneration in Niemann-Pick type C disease from global gene expression profiling. PLoS ONE 1, e19 17183645 10.1371/journal.pone.0000019 PMC1762405

[b79-0061089] Reid P. C. Sugii S. Chang T. Y. (2003). Trafficking defects in endogenously synthesized cholesterol in fibroblasts, macrophages, hepatocytes, and glial cells from Niemann-Pick type C1 mice. J. Lipid Res. 44, 1010–1019 12611909 10.1194/jlr.M300009-JLR200

[b80-0061089] Sagiv Y. Hudspeth K. Mattner J. Schrantz N. Stern R. K. Zhou D. Savage P. B. Teyton L. Bendelac A. (2006). Cutting edge: impaired glycosphingolipid trafficking and NKT cell development in mice lacking Niemann-Pick type C1 protein. J. Immunol. 177, 26–30 16785493 10.4049/jimmunol.177.1.26

[b81-0061089] Sakamoto H. Ukena K. Tsutsui K. (2001). Effects of progesterone synthesized de novo in the developing Purkinje cell on its dendritic growth and synaptogenesis. J. Neurosci. 21, 6221–6232 11487645 10.1523/JNEUROSCI.21-16-06221.2001 PMC6763166

[b82-0061089] Sarna J. R. Larouche M. Marzban H. Sillitoe R. V. Rancourt D. E. Hawkes R. (2003). Patterned Purkinje cell degeneration in mouse models of Niemann-Pick type C disease. J. Comp. Neurol. 456, 279–291 12528192 10.1002/cne.10522

[b83-0061089] Saxena S. Caroni P. (2011). Selective neuronal vulnerability in neurodegenerative diseases: from stressor thresholds to degeneration. Neuron 71, 35–48 21745636 10.1016/j.neuron.2011.06.031

[b84-0061089] Schwend T. Loucks E. J. Snyder D. Ahlgren S. C. (2011). Requirement of Npc1 and availability of cholesterol for early embryonic cell movements in zebrafish. J. Lipid Res. 52, 1328–1344 21576600 10.1194/jlr.M012377 PMC3122913

[b85-0061089] Sleat D. E. Wiseman J. A. El-Banna M. Price S. M. Verot L. Shen M. M. Tint G. S. Vanier M. T. Walkley S. U. Lobel P. (2004). Genetic evidence for nonredundant functional cooperativity between NPC1 and NPC2 in lipid transport. Proc. Natl. Acad. Sci. USA 101, 5886–5891 15071184 10.1073/pnas.0308456101 PMC395893

[b86-0061089] Sleat D. E. Wiseman J. A. Sohar I. El-Banna M. Zheng H. Moore D. F. Lobel P. (2012). Proteomic analysis of mouse models of Niemann-Pick C disease reveals alterations in the steady-state levels of lysosomal proteins within the brain. Proteomics 12, 3499–3509 23070805 10.1002/pmic.201200205 PMC3651901

[b87-0061089] Smith M. M. Levitan D. J. (2007). Human NPC1L1 and NPC1 can functionally substitute for the ncr genes to promote reproductive development in C. elegans. Biochim. Biophys. Acta 1770, 1345–1351 17662536 10.1016/j.bbagen.2007.06.004

[b88-0061089] Somers K. L. Royals M. A. Carstea E. D. Rafi M. A. Wenger D. A. Thrall M. A. (2003). Mutation analysis of feline Niemann-Pick C1 disease. Mol. Genet. Metab. 79, 99–103 12809639 10.1016/s1096-7192(03)00074-x

[b89-0061089] Tang Y. Leao I. C. Coleman E. M. Broughton R. S. Hildreth J. E. (2009). Deficiency of niemann-pick type C-1 protein impairs release of human immunodeficiency virus type 1 and results in Gag accumulation in late endosomal/lysosomal compartments. J. Virol. 83, 7982–7995 19474101 10.1128/JVI.00259-09 PMC2715784

[b90-0061089] Tollervey J. R. Curk T. Rogelj B. Briese M. Cereda M. Kayikci M. König J. Hortobágyi T. Nishimura A. L. Zupunski V. (2011). Characterizing the RNA targets and position-dependent splicing regulation by TDP-43. Nat. Neurosci. 14, 452–458 21358640 10.1038/nn.2778 PMC3108889

[b91-0061089] Tye K. M. Deisseroth K. (2012). Optogenetic investigation of neural circuits underlying brain disease in animal models. Nat. Rev. Neurosci. 13, 251–266 22430017 10.1038/nrn3171 PMC6682316

[b92-0061089] Van Den Eeden S. K. Tanner C. M. Bernstein A. L. Fross R. D. Leimpeter A. Bloch D. A. Nelson L. M. (2003). Incidence of Parkinson’s disease: variation by age, gender, and race/ethnicity. Am. J. Epidemiol. 157, 1015–1022 12777365 10.1093/aje/kwg068

[b93-0061089] Vanier M. T. (2010). Niemann-Pick disease type C. Orphanet J. Rare Dis. 5, 16 20525256 10.1186/1750-1172-5-16 PMC2902432

[b94-0061089] Wöhlke A. Philipp U. Bock P. Beineke A. Lichtner P. Meitinger T. Distl O. (2011). A one base pair deletion in the canine ATP13A2 gene causes exon skipping and late-onset neuronal ceroid lipofuscinosis in the Tibetan terrier. PLoS Genet. 7, e1002304 22022275 10.1371/journal.pgen.1002304 PMC3192819

[b95-0061089] Wong P. C. Cai H. Borchelt D. R. Price D. L. (2002). Genetically engineered mouse models of neurodegenerative diseases. Nat. Neurosci. 5, 633–639 12085093 10.1038/nn0702-633

[b96-0061089] Xu Y. H. Sun Y. Ran H. Quinn B. Witte D. Grabowski G. A. (2011). Accumulation and distribution of α-synuclein and ubiquitin in the CNS of Gaucher disease mouse models. Mol. Genet. Metab. 102, 436–447 21257328 10.1016/j.ymgme.2010.12.014 PMC3059359

[b97-0061089] Yu T. Lieberman A. P. (2013). Npc1 acting in neurons and glia is essential for the formation and maintenance of CNS myelin. PLoS Genet. 9, e1003462 23593041 10.1371/journal.pgen.1003462 PMC3623760

[b98-0061089] Yu W. Gong J. S. Ko M. Garver W. S. Yanagisawa K. Michikawa M. (2005). Altered cholesterol metabolism in Niemann-Pick type C1 mouse brains affects mitochondrial function. J. Biol. Chem. 280, 11731–11739 15644330 10.1074/jbc.M412898200

[b99-0061089] Yu T. Shakkottai V. G. Chung C. Lieberman A. P. (2011). Temporal and cell-specific deletion establishes that neuronal Npc1 deficiency is sufficient to mediate neurodegeneration. Hum. Mol. Genet. 20, 4440–4451 21856732 10.1093/hmg/ddr372 PMC3196892

